# Effect of Copper and Selenium Supplementation on the Level of Elements in Rats’ Femurs under Neoplastic Conditions

**DOI:** 10.3390/nu14061285

**Published:** 2022-03-18

**Authors:** Dorota Skrajnowska, Agata Jagielska, Anna Ruszczyńska, Jakub Idkowiak, Barbara Bobrowska-Korczak

**Affiliations:** 1 Department of Bromatology, Faculty of Pharmacy, Medical University of Warsaw, Banacha 1, 02-097 Warsaw, Poland; dorota.skrajnowska@wum.edu.pl; 2 Biological and Chemical Research Centre, Faculty of Chemistry, University of Warsaw, Zwirki i Wigury 101, 02-089 Warsaw, Poland; ajagielska@chem.uw.edu.pl (A.J.); aruszcz@chem.uw.edu.pl (A.R.); 3 Department of Analytical Chemistry, Faculty of Chemical Technology, University of Pardubice, Studentská 573, 53210 Pardubice, Czech Republic; jakubidkowiak1@gmail.com

**Keywords:** selenium, copper, cancer, supplementation, elements

## Abstract

A study was conducted to determine the effect of long-term supplementation with selenium and copper, administered at twice the level used in the standard diet of rats, on the content of selected elements in the femoral bones of healthy rats and rats with implanted LNCaP cancer cells. After an adaptation period, the animals were randomly divided into two experimental groups. The rats in the experimental group were implanted with prostate cancer cells. The rats in the control group were kept in the same conditions as those in the experimental group and fed the same diet, but without implanted cancer cells. The cancer cells (LNCaP) were intraperitoneally implanted in the amount of 1 × 10^6^ (in PBS 0.4 mL) at the age of 90 days. The content of elements in the samples was determined by a quadrupole mass spectrometer with inductively coupled plasma ionization (ICP-MS). In the femoral bones of rats with implanted LNCaP cells, in the case of the standard diet and the copper-enriched diet, there was a marked decreasing trend in the content of the analysed elements relative to the control rats. This may indicate slow osteolysis taking place in the bone tissue. Contrasting results were obtained for the diet enriched with selenium; there was no significant reduction in the level of these elements, and there was even an increase in the concentrations of Fe and K in the bones of rats with implanted LNCaP cells. Particularly, numerous changes in the mineral composition of the bones were generated by enriching the diet with copper. The elements that most often underwent changes (losses) in the bones were cobalt, iron, manganese and molybdenum. The changes observed, most likely induced by the implantation of LNCaP cells, may indicate a disturbance of mineral homeostasis.

## 1. Introduction

Bone tissue, apart from its support and motor functions and protection of internal organs, plays an important role in metabolic changes in the body, especially mineral metabolism and hematopoietic processes [1,2]. In normal conditions, the entire human skeleton undergoes remodelling over the course of about 5–10 years [3,4]. Bone remodelling takes place due to the metabolic activity of osteoblasts, osteocytes, and osteoclasts [5,6]. The process is hormonally regulated and depends on the presence of essential mineral elements. The content of structural, trace, and toxic elements in the bones and their correlations are also associated with the effect of environmental factors, diet, and disease [2,7,8,9,10,11,12]. Our study analysed supplementation of the diet of rats with minerals that play an important role in the functioning of the body, the effects of which are very closely associated with the dose and degree of saturation. We analysed the effect of a chronic intake of selenium and copper in the diet, at double the level used in the standard (unsupplemented) diet, on the content of selected minerals in the femoral bone of healthy rats and rats with implanted LNCaP prostate cancer cells. The choice of these two elements was based on the assumption that they can be considered in the context of anti- and pro-cancerous effects as well as their significant role in bone metabolism [13,14,15,16,17,18,19]. Copper is a cofactor for the enzyme lysyl oxidase, responsible for collagen crosslinking. Impairment of the activity of this enzyme leads to a weakening of the bones. Moreover, at appropriate concentrations copper indirectly scavenges free radicals as a cofactor of superoxide dismutase, and also directly inhibits activity of osteoclasts [13,20,21]. On the other hand, excess copper can reduce lipid metabolism and also generate lipid peroxidation and interfere with bone metabolism by producing excess amounts of free radicals [14,22,23]. Copper deficiency can also lead to bone developmental defects, hypoplasia, increased fragility due to decreased strength, impaired bone formation, changes in cartilage, and increased risk of osteoporosis in elderly people [14]. In addition, studies on genetic diseases such as Menkes syndrome and Wilson disease, associated with severe copper deficiency and severe copper toxicity, respectively, have made it possible to explain disturbances in processes involving copper, in both bone tissue (osteomalacia, osteoporosis, and cartilage damage) and other tissues [24].

Copper also plays an important role in angiogenesis, including tumour angiogenesis, by attaching to proangiogenic growth factors and increasing their affinity for endothelial cells (as in the case of angiogenin), regulating the secretion of angiogenic cytokines (FGF1 and IL-1α), and inducing expression of angiogenic growth factors (e.g., VEGF) [15,25,26,27,28].

Selenium, as a component of selenoproteins functioning as antioxidants, significantly influences bone metabolism, partially due to its important role in the proliferation and differentiation of osteoclasts or osteoblasts, enhancing the differentiation of osteoblasts by reducing free radicals [16,29,30]. Bone remodelling partially depends on a controlled amount of reactive oxygen species (ROS). An excess of ROS inhibits the differentiation of osteoblasts of bone marrow stem cells (BMSC) and stimulates the differentiation and formation of mature osteoclasts [16,31,32]. This takes place through various mechanisms. The main pathway regulating the balance between osteoblasts and osteoclasts is the RANK/RANKL/OPG system (receptor activator of nuclear factor κB/receptor activator of nuclear factor κB ligand/osteoprotegerin) [33]. Osteoprotegerin is a protein belonging to the family of tumour necrosis factor receptors (TNFR). RANKL belongs to the family of tumour necrosis factors (TNF) [34]. RANKL is produced by mature osteoblasts and their precursors and by activated T cells. It is a factor activating the process of formation of mature osteoclasts and a further cascade of signals essential for their differentiation, fusion, functioning, and survival. RANK ligand acts through the RANK receptor located on the surface of osteoclasts [34,35,36]. RANK ligand is strongly expressed on osteoblasts, osteoclasts, primary mesenchymal cells surrounding cartilage, chondrocytes, endothelial cells, activated T cells, and immature thymocytes (CD4/CD8) [34,36]. Osteoprotegerin is capable of binding to RANKL (as a soluble receptor), which prevents RANKL from binding to RANK. By binding RANKL, OPG blocks maturation, activates osteoclasts, and increases their apoptosis [35,36,37]. The balance between RANKL and OPG regulates the development and activation of osteoclasts, thereby regulating bone metabolism. Differentiation of osteoclasts can be regulated through activation of the receptor activator of nuclear factor κB ligand (RANKL) or macrophage colony-stimulating factor (M-CSF). Administration of Se has been shown to inhibit RANKL-induced gene expression and phosphorylation of IκBα inhibitor, decreasing ROS production and silencing the osteoclast differentiation signal [16,37]. Selenium may protect bone marrow stem cells from H_2_O_2_-induced inhibition of osteoblast differentiation, including through activation of the signalling pathway of ERKs (extracellular signal-regulated kinases), which are responsible for regulating cell proliferation and differentiation [29]. In addition, in conditions of low Se concentrations, osteoblasts show reduced expression of selenoproteins, especially glutathione peroxidases (GPX) and thioredoxin reductases (TRR), and chromosome damage may occur, leading to the appearance of micronuclei [38].

Selenium deficiency in the diet of animals leads to a decrease in bone mineral density (BMD) and bone volume by increasing bone resorption and microarchitectural changes [39]. In humans, the selenium level is inversely correlated with bone remodelling and positively correlated with BMD, especially in postmenopausal women with euthyreosis [17] and in healthy elderly men [19]. Adequate selenium intake has been inversely correlated with a decreased risk of osteoporotic femoral neck fracture [40]. Selenium deficiency and gene polymorphisms responsible for this element can lead to Kashin–Beck disease, which causes impairment of bone growth and degenerative joint diseases [41,42,43,44]. Selenoproteins expressed in human foetal osteoblasts are believed to protect bone against oxidative stress, which can contribute to the development of osteoporosis in later life, mainly by inhibiting the differentiation of osteoblasts of bone marrow stem cells. Many studies have attempted to verify the link between selenium in the diet or serum and bone mineral density (BMD), osteoporosis, or osteoporotic fractures [45]. Of 10 representative studies on this subject, 2 showed that selenium in the serum and diet are probably positively correlated with BMD [19,46] and 3 found that dietary selenium is negatively associated with osteoporotic fractures [40,47,48]. One study found no link between dietary selenium and BMD [49] and the other four found that neither dietary nor serum selenium is correlated with osteoporosis [50,51,52,53]. The authors of the meta-analysis of these studies stressed that their conflicting results may be due to factors such as differences in the study design. It remains unclear whether selenium content in the diet and in the body can directly modulate BMD and influence the pathogenesis of osteoporosis [45].

Another meta-analysis investigated whether there was a correlation linking bone condition with selenium concentration in the blood, selenium intake with meals, and the use of selenium supplements. The analysis included 21 studies: 10 cross-sectional studies; 6 case–control studies; 2 randomized, double-blind, placebo-controlled studies; 2 cohort studies; and 1 longitudinal study [33]. Most of the studies showed a positive link between selenium and bone health, BMD, and a reduced risk of osteoporotic fractures. However, two large randomized studies found that selenium supplementation caused no significant changes [54]. In the first study, conducted only in post-menopausal women, this finding may have been due to a higher initial dietary selenium intake. In the second study, conducted in older people with reduced selenium levels, measurement of bone turnover markers after 6 months showed a significant linear decrease in the level of procollagen type 1 *N*-terminal propeptide (P1NP), which indicates reduced bone formation. No significant effect of selenium supplementation on other bone formation markers was observed. Hence, confirmation of a significant effect of selenium on bone health requires further randomized clinical studies [55].

There are many hypotheses regarding the potential anticarcinogenic mechanism of the action of selenium. The strongest positive effect may be the antioxidant activity of glutathione peroxidase and selenoprotein P [18,56]. Selenium plays a role in the regulation of protein folding, mediated by its effect on the necrosis and apoptosis of the endoplasmic reticulum [57]. Apoptosis is the process of programmed suicidal cell death. It takes place via several pathways, such as the endoplasmic reticulum stress pathway [58,59]. Necrosis, on the other hand, is induced by external factors, mainly physical (e.g., low or high temperature or radiation), and mechanical factors, which degrade cells [60]. Selenium also exerts a stabilizing effect on DNA [18]. An inverse relationship was noted between the selenium concentration in the blood and the risk of prostate cancer [18] and selenium was shown to inhibit the proliferation of prostate cancer cells stimulated by exposure to cadmium [61,62]. However, the effect of selenium is highly subject to the dose–response relationship [61]. By oxidizing or reducing external –SH groups and disulphide bridges of growth factors and functional proteins, selenium functions as a redox switch. However, for this purpose, it must reach a concentration for which a further increase allows it to initiate apoptosis, e.g., by DNA-fragmenting methyl selenol (CH3SeH, a metabolite of selenium compounds) [18,63]. The capacity of selenium to cause the death of cancer cells is more pronounced in the case of androgen-dependent lines. Androgens cause multiplication and oxidation in all prostate cells, healthy and cancerous. If selenium compounds reach a sufficient concentration, they can inhibit the G1, G2, and S phase of the cell cycle, thereby preventing tumour formation [64,65,66]. Selenium can also directly react with carcinogens (e.g., DMBA or aflatoxins), preventing them from binding to DNA [67]. Many studies are conducted on the relationships between selenium and cancer risk. Epidemiological studies and randomized clinical trials show varied effects in humans. Preventive and therapeutic effects of selenium, as well as carcinogenic effects, have been hypothesized. The authors of several studies have reported a correlation between high selenium concentrations in the blood and the appearance of cancer [18,68]. Some studies on breast cancer have demonstrated a protective effect of selenium, while others have shown no association [18,69].

The materials for our study were the femoral bones of rats, because prostate cancer shows a particularly strong tendency to form metastatic foci in this tissue [7,70]. Bone metastases are usually located in the spine, ribs, pelvis, and femoral and humeral bones, where they disturb physiological bone processes, haematopoiesis, and the immune system [71]. Two basic types of metastatic changes in the skeleton are distinguished: osteolytic (stimulation of osteoclasts) and osteoblastic (stimulation of osteoblasts). Osteoblastic changes can take place in the bones due to the production of fibroblasts, insulin-like and vascular endothelial growth factors (FGF, IGF, and VEGF), and endotheline-1 by cancer cells [72]. Cancer cells involved in metastasis to the bones involve the host microenvironment in this process. A well-known consequence of osteolytic metastases in the bones is hypercalcaemia, caused by the release of calcium stored in the bones [73,74,75]. Do other trace minerals forming bone tissue undergo changes, and to what extent?

The aim of the study was to identify differences in the mineral composition of the bones of rats with implanted LNCaP prostate cancer cells and to determine whether specific modification of the diet, involving the use of additional copper and selenium ions, would affect the content of elements in the bones.

## 2. Materials and Methods

### 2.1. Ethics Approval Statement

This research and guiding principles in the care and use of laboratory animals were approved by the 2nd Local Ethical Committee on Animal Experiments at the Medical University of Warsaw.

### 2.2. Dietary Ingredients

Labofeed H feed, used in this research, is intended for adult rats and provides them with optimal conditions for growth and reproduction. Laboratory fodder Labofeed H was purchased from the Morawski Feed and Concentrates Production Plant (Kcynia, Poland).

The diet contained the following compounds (per 1 kg): protein (210 g), fat (39.2 g), fibre (43.2 g), ash (55 g), carbohydrates (300 g), vitamin A (15,000 IU), vitamin D3 (1000 IU), vitamin E (90 mg), vitamin K3 (3 mg), vitamin B1 (21 mg), vitamin B2 (16 mg), vitamin B6 (17 mg), vitamin B12 (80 µg), pantothenic acid (30 mg), folic acid (5 mg), nicotinic acid (133 mg), Ca (10.0g), P (8.17 g), Mg (3 g), K (9.4 g), Na (2.2 g), Cl (2.5 g), S (1.9 g), Fe (250 mg), Mn (100 mg), Zn (76.9 mg), Co (2.0 mg), I (1.0 mg), Cu (21.3 mg), and Se (0.5 mg).

### 2.3. Animal Experiments and Experimental Procedure

Forty-one healthy male Sprague-Dawley rats were obtained from the authorized animal care unit of the Animal Laboratory, Department of General and Experimental Pathology from the Medical University of Warsaw. The animals were housed under the standard conditions at 22 °C, the relative humidity of 55%, and 12-h light–dark cycle. They had free access to food (standard diet: Labofeed H) and deionized water (from Milli-Q System (Merck, Millipore, Germany)). The experiment was conducted over 90 days. Before the start of the experiment, animals were kept in the same cages 10 days for acclimatization (10 days—rats’ age 60 to 70 days). After the adaptation period, the animals were randomly divided into three dietary groups: standard diet and supplementation with Cu or Se. Every dietary group was divided into the experimental group (with implanted cancer cells—Exp) and the control group (without implanted cancer cells—Ctrl). The LNCaP prostate cancer cells were injected intraperitoneally, in the amount of 1 × 10^6^ (in PBS 0.4 mL), into the rats at day 90 of their lifetime. The certified line of androgen-dependent human prostate cancer cells was obtained from ATCC bank (American Type Culture Collection, Menassas, VA, USA). LNCaP cells were maintained in DMEM medium supplemented with 10% (*v*/*v*) FBS, sodium pyruvate (1 mM), penicillin (100 U/mL), and streptomycin (100 μg/mL) at 37 °C in a 5% CO_2_ humidified atmosphere.

The rats were fed extra supplements suspended in deionized water, 0.4 mL daily, from 70 days until 150 days of age, when they were sacrificed by decapitation. The animals that were fed only the standard diet (without supplementation) received 0.4 mL of water. The doses of trace elements were selected based on the values used in the Labofeed H diet. According to the level of trace elements in the Labofeed diet, the rats were fed, via gavage, extra supplements of the following: double dose of Cu and Se.

The animals from both groups—experimental (Exp) and control (Ctrl)—were provided with the minerals by oral gavage, in a solution:

- Copper—0.639 mg/mL (0.256 mg Cu(II)/day/rat, as CuSO_4_·5H_2_O in aqueous suspension);
- Selenium—0.018 mg/mL (0.0072 mg Se(VI)/day/rat, as Na_2_SeO_4_ in aqueous suspension).

The materials for the study were rat femoral bones. Following resection, the adjacent soft tissues, i.e., the joint capsule and muscle, were removed from the bones, and then they were frozen at −80 °C. Immediately before analysis, the bones were thawed, dried for 10 h at 120 °C, and mineralized in a 5 mL 65% HNO_3_ solution (Plazmatronika mineralizer). Then, deionized water was added to the digest solution to a volume of 10 mL. The content of ten elements (Ca, K, Fe, Sr, Zn, Ni, Cu, Mn, Co and Mo) was determined in the digest solutions by inductively coupled plasma mass spectrometry (ICP-MS), using the following dilutions:

(1) 5 fold for determination of Co, Cu, Mn, Mo, Ni, Se and Zn;
(2) 500 fold for determination of Ca, K, Sr and Fe.

#### 2.3.1. Chemicals and Reagents

Calibration solutions were prepared daily from ICP multi-element standard Merck VI (Merck, Darmstadt, Germany). Required dilutions of standard and samples after digestion were obtained by using deionized water from Milli-Q System (Merck, Millipore, Germany).

#### 2.3.2. Analytical Procedure

The content of elements in the samples was determined with the 5-point calibration curve method (standards from 1 μg/L to 100 μg/L for Mg, K, Mn, Co, Cu, Ni, Sr, and Mo; standards from 10 μg/L to 1000 μg/L for Fe, Se, and Zn; standards from 100 μg/L to 10,000 μg/L for Ca).

#### 2.3.3. Instrumentation

Quadrupole mass spectrometer with inductively coupled plasma ionization, ICP-MS, (Nexion 300D, Perkin Elmer Sciex, 940 Winter Street Waltham, MA 02451, USA) was used. Solutions were directly introduced into the Meinhard nebulizer and quartz cyclonic spray chamber. The spectrometer working conditions (Table 1) were verified daily and optimized in order to obtain the lowest level of oxides and double charged ions as well as the maximal sensitivity of isotopic detection.

### 2.4. Statistics

Student’s t-test was used to compare the content of individual elements in the control vs. experimental groups, separately for each diet. Differences between groups were considered significant at *p* < 0.05. For the comparison of all groups, first the Kruskal–Wallis test was performed. At each step, all obtained p-values were corrected using the Benjamini–Hochberg FDR approach. Statistical tests were performed in R version 4.1.2 (https://www.r-project.org/ (accessed on 1 January 2022)), using rstatix 0.7.0 library (https://rpkgs.datanovia.com/rstatix/ (accessed on 1 January 2022)). Box plots were generated using ggplot2 version 3.3.5 (https://ggplot2.tidyverse.org/ (accessed on 1 January 2022)) and ggpubr version 0.4.0 libraries (https://rpkgs.datanovia.com/ggpubr/ (accessed on 1 January 2022)), for box plots with dot plots. PCA analysis was performed using the MetaboAnalyst 5.0 platform (https://www.metaboanalyst.ca/ (accessed on 1 January 2022 )) and the data were log-transformed and Pareto-scaled before the PCA analysis.

## 3. Results

### 3.1. Experimental to Control Group Comparison

Mean values with standard deviations for the content of 10 elements (Ca, K, Fe, Sr, Zn, Ni, Cu, Mn, Co, and Mo) in the bone of rats are presented in Table 2, separated for different diet groups and for different treatment groups: Exp (experimental with implanted cancer cells LNCaP) and Ctrl (control without implanted cells LNCaP).

The experimental-to-control ratios (%) for the content of elements indicate a downward trend in most cases. The reverse tendency was noted only in the group receiving a selenium supplement, in which the content of two elements (K and Fe) increased in the bones of rats with LNCaP implantation (by 42% and 43%, respectively). An especially large number of differences in the mineral composition of the bones between the groups with and without implanted LNCaP were generated by the copper-supplemented diet. In the group of rats receiving this diet, there were significant reductions in the content of Mo (by 69%), Co (59%), Mn (54%), Cu (61%), Ni (50%), Zn (32%), and Fe (33%) in the bone tissue of rats with implanted LNCaP cells in comparison with the control group receiving the same diet, but without LNCaP implantation. There were no changes in the concentration of Ca, K, and Sr (Table 2).

In the group of rats receiving the standard diet (without supplementation) there was a statistically significant reduction in Ca (by 24%), Sr (9%), Mn (13%), Co (22%), and Mo (83%) in the bone tissue of rats with implanted LNCaP cells in comparison to the control group that also received the standard diet (Table 2).

### 3.2. Comparison of Diet Groups

#### 3.2.1. Dietary Supplementation with Copper Relative to Other Experimental Groups

**Figure 1:**
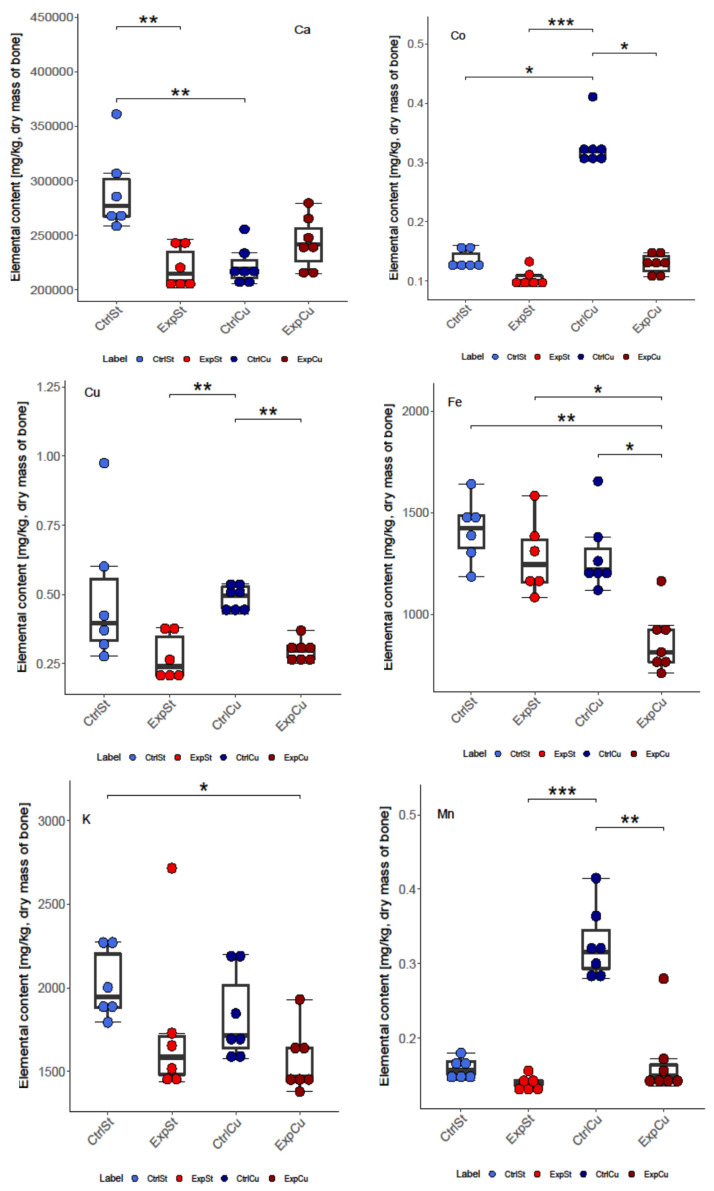

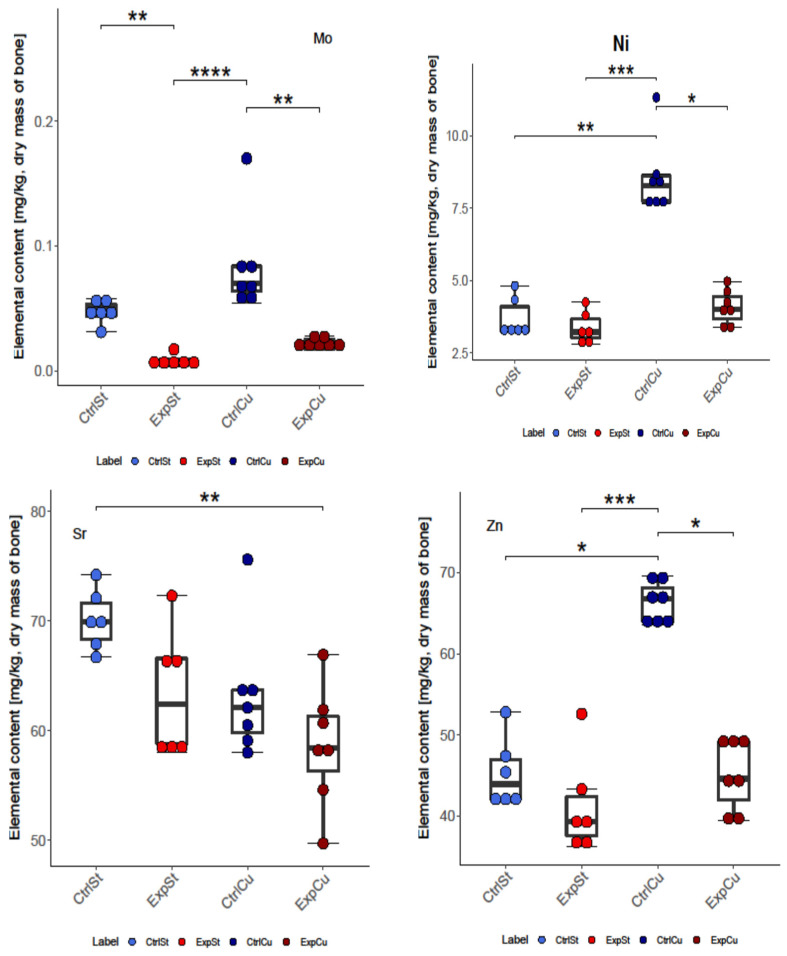
Analysis of concentrations of elements in four experimental groups: CtrlSt—control standard diet without LNCaP (blue boxplot); ExpSt—experimental standard diet with LNCaP (red boxplot); CtrlCu—Cu supplementation diet without LNCaP (dark blue boxplot); ExpCu—Cu supplementation diet with LNCaP (dark red boxplot); *p*-value: <0.0001 ****, 0.0001–0.001 ***, 0.001–0.01 **, 0.01–0.05 *.

In the case of copper supplementation, changes were noted in the bones of rats for all 10 elements analysed, i.e., Ca, Co, Fe, Cu, K, Zn Mo, Mn, Sr, and Ni. The following statistically significant relationships were obtained (Figure 1):

- The greatest number of statistically significant differences between nearly all groups was shown for the levels of Fe, Co, Mo, Ni, and Zn. It is particularly worth noting the significant decrease in the content of these elements in the groups with implanted LNCaP cells (ExpCu) in comparison with the control group (CtrlCu) without implanted cancer cells.
- In the case of Ca and Mo, a decrease was noted in the experimental groups whose diet was not supplemented (ExpSt) relative to the corresponding control group (CtrlSt). Comparison of the control groups with and without copper supplementation (CtrlCu vs. CtrlSt) also showed a decrease in Ca levels, but an increase in the content of Co in the control group receiving additional copper (CtrlCu).
- Comparison of groups ExpSt and CtrlCu showed an increase in Co, Mn, Mo, Ni, and Zn levels in the control group. Comparison of ExpCu vs. CtrlSt revealed a decrease in K, Sr, and Fe in group ExpCu.
- Single changes were observed for K and Sr.

**Figure 2:**
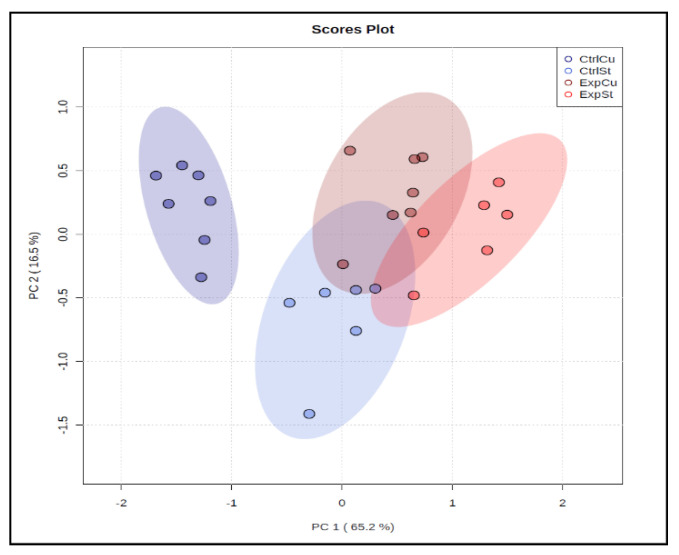
The principal component analysis performed on four groups: rats on the standard diet with LNCaP (red dots—ExpSt); rats on the standard diet without LNCaP (blue dots—CtrlSt); rats with Cu supplementation with LNCaP (dark red dots—ExpCu); rats with Cu supplementation without LNCaP (dark blue dots—CtrlCu).

In the multivariate analysis, principal component analysis (PCA) separated one group (CtrlCu) from the other three, showing that copper supplementation in rats without LNCaP implantation had a clear influence on bone composition (Figure 2).

#### 3.2.2. Dietary Supplementation with Selenium Relative to Other Experimental Groups

**Figure 3:**
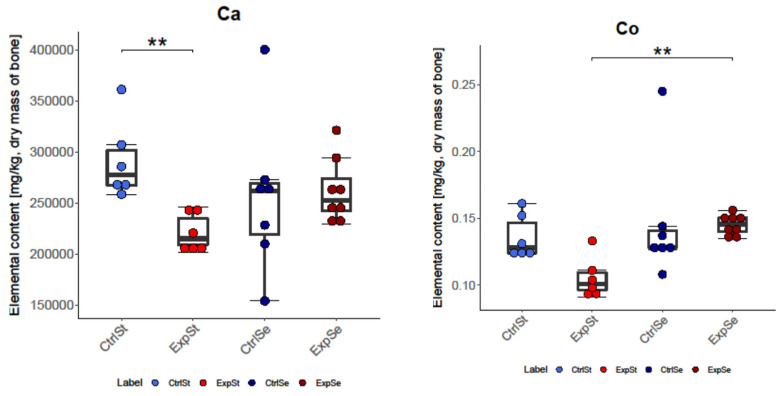

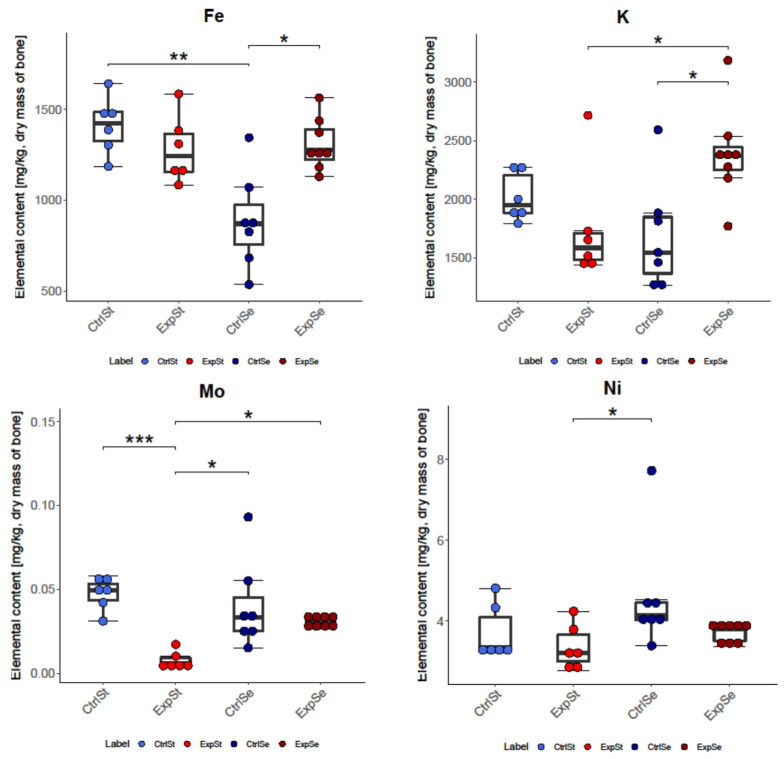
Analysis of concentrations of elements in four experimental groups: CtrlSt—control standard diet without LNCaP (blue boxplot); ExpSt—experimental standard diet with LNCaP (red boxplot); CtrlSe—Se supplementation diet without LNCaP (dark blue boxplot); ExpSe—Se supplementation diet with LNCaP (dark red boxplot); *p*-value: 0.0001–0.001 ***, 0.001–0.01 **, 0.01–0.05 *.

In the case of selenium supplementation, changes in the bones were observed for six elements: Ca, Co, Fe, K, Mo, and Ni. The following statistically significant relationships were obtained (Figure 3):

- The greatest number of changes was noted for Mo. There was a significant decrease in its content in group ExpSt relative to groups CtrlSt, CtrlSe, and ExpSe.
- For iron, there was a decrease in the bones of rats in group CtrlSe relative to groups CtrlSt and ExpSe.
- Potassium content increased in the bones of rats with implanted LNCaP (ExpSe) in comparison to the control group on the same diet (CtrlSe) and ExpSt.
- Single changes were observed for Ca (CtrlSt vs. ExpSt), Co (ExpSt vs. ExpSe), and Ni (ExpSt vs. CtrlSe).

**Figure 4:**
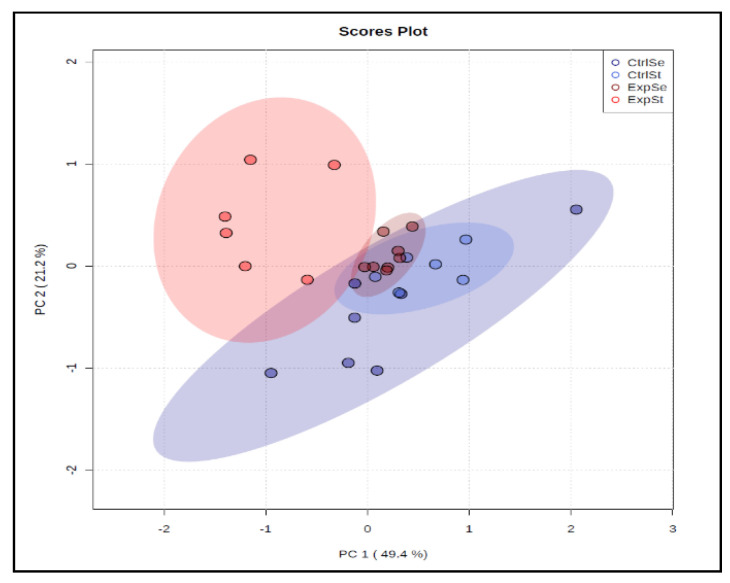
The principal component analysis performed on four groups: rats on the standard diet with LNCaP (dark red dots—ExpSt); rats on the standard diet without LNCaP (red dots—CtrlSt); rats with Se supplementation with LNCaP (dark blue dots—ExpSe); rats with Se supplementation without LNCaP (blue dots—CtrlSe).

Principal component analysis indicated that the presence of cancer cells had a marked effect on the bone composition of rats on the standard diet (ExplSt is separated from the other groups, Figure 4).

## 4. Discussion

The most common prostate diseases, which are primarily diagnosed in older people, are benign prostatic hyperplasia (BPH) and prostate cancer (PCa). BPH is an increase in the volume of the prostate due to cellular proliferation of the prostate tissue surrounding the urethra [76,77,78]. Prostate cancer (PCa) is the second cause of death from oncological diseases in Western countries. In 2020, there were 1,414,259 new cases in men of all ages [79,80]. The disease usually begins asymptomatically in the form of a small focus located only in the outer part of the prostate. Over time, the patient begins to have symptoms, such as frequent urination and difficulty urinating due to proliferation of the cancer and infiltration into the prostate and periprostatic tissue. Subsequently, the cancer cells migrate via the blood and lymph vessels to other tissues, where they form metastases and are the main cause of the death of the patient. Prostate cancer shows a strong tendency to form metastatic foci in the bones, lymph nodes, and lungs [81,82]. However, in most patients the site of tumour invasion is the microenvironment of the bone marrow. Bone is a metabolically active tissue, which means that bones will be affected both by illness and by overall nutritional status. The organic matrix of bone consists of osteoclasts, osteoblasts, osteocytes, scleroproteins, globular proteins (osteonectin and osteocalcin), and substances such as proteoglycans and glycoproteins. The inorganic matrix is mainly hydroxyapatite (calcium, phosphorus and magnesium), as well as small quantities of trace elements (Fe, Zn, Cu, B, Se, As, W, U, Ti, Sr, Si, Na, K, Mo, Pt, Hg, Mn, Li, Ge, Ga, F, Co, Cd, and Al), which can be essential or toxic depending on their concentration [14]. Skeletal tissue, like the entire body, is constantly undergoing changes, in this case through resorption by osteoclasts and the formation of new cells by osteoblasts. In addition to macroelements such as calcium, phosphorus and magnesium, which play an important and well-known role in bone formation, trace elements also affect bone metabolism [83]. These elements can be incorporated into the bone mineral matrix, influence the proliferation or activity of osteoblasts or osteoclasts, and serve as cofactors for key enzymes taking part in the mineralization of bone tissue. Noor et al. [84] showed that the concentrations of B, Al, S, V, Co, Mo, Te, Ba, La, Ni, and As in osteoporosis are elevated relative to healthy bones. In contrast, the concentrations of Na, Mg, P, K, Ca, Cr, Pd, Ag, Mn, Fe, Cu, Zn, Rb, Sr, Pb, and Se were lower than in the bone tissue of healthy subjects.

Our study showed that implantation of cancer cells clearly alters the content of elements in the femoral bones of rats fed a standard diet, and that enrichment of the diet with selected elements modifies these changes. Thus, supplementation of the diet of rats with selenium in conditions of implantation of prostate cancer cells caused a marked decrease in the frequency of changes in the mineral composition of the bone tissue, while supplementation with copper markedly increased the differences in the content of these elements in conditions of implantation of prostate cancer cells.

Copper has a very important role in the skeletal system. Many studies have confirmed that Cu deficiency impairs the mechanical strength of bones by reducing the crosslinking of elastin and collagen. Collagen cross-linking disorders have been correlated with impaired activity of lysyl oxidase, which forms crosslinks derived from lysine. Cu deficiency also decreases the activity of superoxide dismutase, thereby increasing the activation of osteoclasts and bone resorption [83,85,86]. In vitro studies have shown a positive effect of copper on cells regulating bone metabolism, e.g., by inhibiting osteoclast resorption [21]. Other authors emphasize the effect of the dose. Thus, low Cu concentrations (0.1% *w*/*w*) improved the viability and growth of osteoblastic cells, whereas higher concentrations (2.5% and 1% (*w*/*w*)) proved to be cytotoxic [87]. In our study, we used a copper dose amounting to double the level used in the standard diet. Parameters such as body and organ weight, appetite, and the general condition of animals, as well as the state of the hair and nails, were satisfactory. The content of minerals in the diet of laboratory animals depends on their age, physiological condition, and the purpose of the experiment (surplus or deficiency). In accordance with recommendations by the Federation of European Laboratory Animal Science Associations, rats’ requirements for individual nutrients is based on their content in feed [88]. The feed used in our study, Labofeed H, is meant for adult rats and provides them with optimal conditions for growth and reproduction. The content of Cu and Se is 21.3 mg and Se 0.5 mg/kg feed, respectively. Additional supplementation in individual groups allowed for total intake of 63.9 mg Cu and 1.5 mg Se/kg feed. Pathological changes in the liver and kidneys appear when diets contain more than 1.000 mg/kg Cu, and significant weight loss is observed in the case of diets containing 2.000 mg Cu/kg [88]. Therefore, it seems that the considerable loss of minerals from the bones could not have been caused by copper supplementation alone, especially since a similar effect was noted in the case of the standard diet. It was most likely the implantation of LNCaP cancer cells that induced the changes in the femoral bone tissue, but copper supplementation generated the most changes in mineral composition; in addition to molybdenum, these also included reductions in zinc, iron, nickel, manganese, and copper. It should also be noted that the elements that most often underwent changes in our experiment were manganese, molybdenum, cobalt, and iron. Among all elements analysed, the greatest decrease was noted for the concentration of molybdenum in the bones of rats with LNCaP, with (by 69%) and without (83%) copper supplementation. Molybdenum is an essential trace element that serves as a cofactor for several redox enzymes, but its physiological role is largely unknown [89], and studies evaluating the relationship between biomarkers of exposure to Mo and the state of bones are lacking [90]. Excess copper in the body is known to reduce concentrations of molybdenum, while excess molybdenum reduces the level of copper, increasing the amount excreted in the urine and interfering with its absorption from the gastrointestinal tract. This is because molybdenum in the intestinal lumen creates insoluble complexes with copper (copper molybdate and copper thiomolybdate), thereby preventing its absorption and incorporation into plasma proteins, such as ceruloplasmin and other proteins containing copper [91,92]. There are also reports that Mo exerts an effect on cell proliferation independently of Cu status in an unknown mechanism. Excess Mo with the diet impairs cell proliferation within the growth plate, whereas the effects of copper deficiency are more associated with the differentiation of chondrocytes. In this manner, Mo can induce changes in longitudinal bone growth that differ from those arising from Cu deficiency [93]. Molybdenum deficiency in the early stages of animal development also inhibits growth [14,94]. Antagonism between copper and zinc and synergy between copper and iron are known as well [86]. Manganese has a broad spectrum of action as a cofactor of superoxide dismutase and numerous enzymes involved in the synthesis of cartilage proteoglycans, such as glycosyltransferase, xylosyltransferase, phosphohydrolase, and phosphotransferase [14,95]. Superoxide dismutase, which contains manganese, protects osteoblasts from ROS emitted by osteoclasts [96]. Prolonged deficiency in the bone can lead to abnormalities in bone tissue, impaired osteogenesis, thickening of the bones, epiphyseal dysplasia, and inhibition of bone growth, as well as osteoporosis and chondrodystrophy [84].

The second of the elements named above, i.e., cobalt, can modulate bone metabolism and cause osteolysis, but reports of this type mainly concern cobalt from implants [97]. Cobalt ions have been shown to influence the proliferation, size, and shape of osteoblasts. The pathogenesis of osteolysis involves inhibition of osteoblast function and stimulation of the production and secretion of chemokines (TGF-β1, TNF-α, IL-β 1, IL-6, IL-8, and MCP-1) from the osteoblasts, leading to inflammation and the differentiation, maturation, and stimulation of osteoclasts in the periprosthetic area [97,98,99]. Moreover, cobalt ions significantly inhibit osteoblast function by decreasing the activity of alkaline phosphatase and through calcium deposition [99,100]. Divalent cobalt ions can also probably generate oxidative stress in the osteoblasts, but the mechanism of action is unknown [101,102]. Suggested mechanisms of the toxic effect of cobalt ions include radical generation, impairment of cell membrane function, or inhibition of enzyme function.

Another element whose content decreased in the group of rats receiving a standard diet, or a diet supplemented with copper, was iron. Iron is a cofactor of numerous enzymes, including oxoglutarate-dependent 2-dioxygenase, which plays a key role in collagen synthesis [39,103]. A study in which female rats were fed a diet with severely reduced iron for 5 weeks showed a decrease in the number and thickness of trabeculae and in bone strength, and an increase in trabecular separation. Apart from structural disturbances in the bone tissue, iron deficiency can also affect markers of bone turnover. In a study by Castro et al. [104], female rats were given an iron-poor diet for 40 days. This resulted in a decrease in the level of the bone formation marker procollagen type I N-terminal propeptide (P1NP) relative to the rats on a diet with normal iron levels, accompanied by an increase in the concentration of tartrate-resistant acid phosphatase 5b (TRAP), parathormone, and C-terminal telopeptides (CTX). These findings indicate that severe iron deficiency in humans may result in disorders of bone tissue formation and additionally increase bone resorption [83]. However, moderate iron deficiency is not clearly linked to bone tissue disorders. On the other hand, excessive accumulation of iron in the body is associated with diseases such as haemoglobinopathy, haemochromatosis, or menopause with reduced bone mass, osteopaenia, osteoporosis, altered bone microarchitecture, and elevated fracture risk [105].

The effect of copper supplementation on the condition of bones has been assessed in various studies, mainly in menopausal women [106,107,108,109]. Intake of 3 mg of copper in the diet of healthy women for 2 years decreased vertebral bone density loss relative to the control group without supplementation [107]. Baker et al. [108] studied the effect of a 3-week diet with various levels of copper (1.6 mg/d vs. 0.7 mg/d vs. 6 mg/d) in 11 healthy men. Markers of bone resorption significantly decreased with the transition from a copper-poor to a copper-rich diet. Therefore, it seems that copper in nontoxic doses should not generate osteolytic changes in bone tissue.

A number of studies link Cu toxicity to the formation of reactive oxygen species, which modify the structure and/or function of basic biomolecules. An excess of ROS inhibits the efficiency of antioxidant systems and leads to DNA damage and peroxidation of lipids and proteins. This may result in the development of degenerative diseases, including cancer, cardiovascular disease, diabetes, atherosclerosis, neurological disorders, and chronic inflammation [110]. Patients with advanced metastasizing prostate cancer have been shown to have higher levels of oxidative stress, measured as the degree of susceptibility of serum lipids to peroxidation, in comparison with patients with locally advanced prostate cancer [111]. Oxidative stress follows increased ROS production and/or simultaneous impairment of antioxidant capacity. ROS are constantly generated in healthy cells, and small changes in redox homeostasis are necessary for natural regulation of cellular functions by various mechanisms, such as activation or inactivation of transcription factors, metabolic enzymes, membrane channels, and others [112]. Prostate cancer cells (PC), in comparison to healthy cells, are characterized by innate oxidative stress, which distinguishes the aggressive phenotype of this disease [113]. Characteristic metabolic changes, activation of androgen receptor, and mutation-induced mitochondrial dysfunctions take place, while the effects of external environmental factors and metabolism of xenobiotics inducing inflammation and hypoxia are increased [112]. However, the relationships between oxidative stress, redox homeostasis, and activation of proliferation and survival pathways in the healthy and cancerous prostate are not fully understood.

The other element used to supplement the diet of rats in our study was selenium. Inadequate Se intake can result in increased ROS and oxidative stress, especially in people with low levels of other antioxidants (e.g., vitamins E and C). Selenium is a component of over 25 selenoproteins neutralizing free radicals, but there is a threshold of selenium exposure below and above which no significant anticancer effect is observable [16]. Additionally, different forms of selenium, by acting on different metabolic pathways, can have varied effects. This could explain the discrepancies in the scientific literature, as selenium intake considerably varies depending on geographic location [114]. Meta-analyses of case-control studies have shown an inverse risk of PCa in men with high serum selenium concentrations [115,116]. Other studies have not found that selenium supplementation affects the incidence of PCa [117,118,119]. Taking into account the dose–response relationship of all antioxidant supplements, another meta-analysis concluded that selenium may protect against PCa only in populations with a low baseline serum concentration of selenium.

Selenium plays multiple roles in the cell, influencing the cell cycle, apoptosis, the immune system, and the condition of bones [16,120,121,122]. Its role in bone metabolism is very important. Nine selenoproteins have been shown to be expressed in human foetal osteoblasts, which most likely contributes to protection against oxidative stress in the bone microenvironment [16]. Bone marrow stromal cells (BMSC) cultured in a selenium-deficient medium exhibited decreased expression of selenium-dependent enzymes such as glutathione peroxidase and thioredoxin reductase, as well as chromosome damage, which was later reversed by the addition of selenium [38]. Selenium deficiency in the diet of rats increased bone resorption, not only due to the decrease in the activity of selenium-dependent enzymes, but also to a decrease in calcium concentration, disturbed growth hormone secretion in the pituitary gland, reduced insulin-like growth factor in the plasma, increased concentrations of parathyroid hormone and 1,25-dihydroxyvitamin D, and excessive excretion of calcium in the urine [16,39]. It is very likely that physiological bone remodelling is highly dependent on adequate control of the effects of ROS. The results of our study confirm the protective role of selenium in terms of loss of elements from bone tissue. In animals receiving selenium supplements, fewer changes in the concentrations of elements in the bone were noted during the neoplastic process in comparison with the animals receiving a standard diet or a diet supplemented with copper. However, there were also significant changes involving an increase in potassium and iron content in the tissue of rats with implanted LNCaP in comparison to the group without implantation on the same diet. The reason for this is unclear. Potassium in the body is closely linked to sodium and calcium metabolism. Supplementation with both potassium citrate and calcium citrate can lead to increased absorption of calcium, a reduction in markers of calcium resorption in the urine and bone (C-terminal telopeptide of type 1 collagen and N-terminal telopeptide in the urine), and a decrease in the serum concentration of parathyroid hormone (PTH) [123,124]. However, other studies have found that potassium imbalances do not strongly affect bone metabolism [125]. In our study, the more than 40% increase in potassium content in the bone may indicate progressive disturbances of sodium and calcium metabolism in the body, despite the fact that the calcium content in the bone was not significantly altered in the group receiving the selenium supplement. The more than 40% increase in the iron concentration in the bone is also difficult to explain. Excessive accumulation of iron in various tissues, including bone tissue, is usually caused by genetic disorders, such as haemochromatosis. It leads to increased bone resorption and slows down bone formation. The result is low bone mass, increased frequency of fractures, an overall change in the bone microarchitecture, and increased frequency of osteoporosis [105,126,127,128].

The data presented here show that implantation of LNCaP cancer cells in the conditions of the experiment led to osteolytic changes in the bones, manifested as modified bone tissue composition. Supplementation of the diet of rats with copper probably exacerbated these changes, because losses of Ca, Mn, Sr, Co, and Mo were observed for the standard diet and, additionally, losses of Zn, Fe, Ni, and Cu for the copper diet in the bones of rats with implanted LNCaP relative to the control group. In the case of the diet with additional selenium, no significant deficiencies of elements were noted, but there was a strong increase in the content of potassium and iron, two important elements for bone health. At this stage of research, it is difficult to conclude whether or not this is a beneficial process in tumour development and metastasis. A limitation of our research was the lack of histopathological examination of the bones, but both the mass and the strength of the bone remained unchanged. Metastatic changes usually become visible after a long period, but tumour cells can be initially present in secondary tissue and remain latent, or can be present at the stage of replication and preparation for further expansion. Zeng et al. [16] showed that selenium intake significantly inhibits the osteoblast inflammatory response to metastatic breast cancer cells regulated by NF-kB activation. To conclude, osteolytic changes resulting from metastasis are likely to be slowed down by supplementation with selenium compounds. The optimum chemical form, dosage, and duration of supplementation remain an open question.

## Figures and Tables

**Table 1:** The spectrometer working conditions.

ICP-MS Nexion 300 D	
plasma power, W	1350
nebulizer gas flow (AR), L min^−1^	0.9
dwell time, ms	50
readings	5
sweeps	1
replicates	3
monitored isotopes	^27^Al, ^24^Mg, ^39^K, ^43^Ca, ^51^V, ^55^Mn, ^57^Fe, ^59^Co, ^63^Cu, ^60^Ni, ^66^Zn, ^78^Se, ^88^Sr, ^95^Mo, ^111^Cd, ^208^Pb

**Table 2:** Mean values ± standard deviation and direction and percentage of changes in the elements in the bones of rats with cancer receiving various diets as compared to the control group on the same diet.

Group/ Diet n = 6–8	Ca (g/kg Dry Mass)	Zn (mg/kg Dry Mass)	K (mg/kg Dry Mass)	Fe (mg/kg Dry Mass)	Sr (mg/kg Dry Mass)	Ni (mg/kg Dry Mass)	Cu (mg/kg Dry Mass)	Mn (mg/kg Dry Mass)	Co (mg/kg Dry Mass)	Mo (mg/kg Dry Mass)
CtrlSt ExpSt	291 ± 38	45.3 ± 4.3	2018 ± 206	1411 ± 158	70.1 ± 2.7	3.71 ± 0.69	0.494 ± 0.261	0.160 ± 0.014	0.136 ± 0.016	0.047 ± 0.010
	221 ± 18 *	41.3 ± 6.0	1561 ± 125	1281 ± 184	63.5 ± 5.8 *	3.36 ± 0.55	0.272 ± 0.084	0.140 ± 0.010 *	0.106 ± 0.015	0.008 ± 0.005
	↓ 24%	↓ 9%	↓ 23%	↓ 9%	↓ 9%	↓ 9%	↓ 45%	↓ 13%	↓ 22%	↓ 83%
	(*p* = 0.002)	(ns)	(ns)	(ns)	(*p* = 0.029)	(ns)	(ns)	(*p* = 0.017)	(*p* = 0.007)	*p* = 4.67 × 10^−6^
CtrlCu ExpCu	222 ± 17	66.3 ± 2.6	1827 ± 262	1289 ± 180	61.2 ± 2.4	8.1 ± 0.46	0.488 ± 0.045	0.327 ± 0.048	0.314 ± 0.009	0.070 ± 0.012
	243 ± 23	45.1 ± 4.3 *	1562 ± 191	866 ± 155 *	58.6 ± 5.5	4.07 ± 0.59 *	0.298 ± 0.039 *	0.149 ± 0.013 *	0.129 ± 0.017 *	0.022 ± 0.005 *
	↑ 10%	↓ 32%	↓ 15%	↓ 33%	↓ 4%	↓ 50%	↓ 61%	↓ 54%	↓ 59%	↓ 69%
	(ns)	(*p* = 9.93 × 10^−8^)	(ns)	(*p* = 0.001)	(ns)	*p* = 2.23 × 10^−6^	*p* = 2.23 × 10^−6^	*p* = 2.23 × 10^−6^	*p* = 2.23 × 10^−8^	(*p* = 0.001)
CtrlSe ExpSe	256 ± 76	43.5 ± 4.3	1692 ± 463	887 ± 262	59.6 ± 18.0	4.08 ± 0.4	0.308 ± 0.051	0.179 ± 0.059	0.129 ± 0.012	0.040 ± 0.026
	254 ± 22	45.0 ± 2.1	2402 ± 424 *	1272 ± 106 *	66.7 ± 4.3	3.76 ± 0.21	0.283 ± 0.027	0.157 ± 0.003	0.147 ± 0.007	0.031 ± 0.003
	↓ 1%	↑ 3%	↑ 42%	↑ 43%	↑ 12%	↓ 8%	↓ 8%	↓ 12%	↑ 14%	↓ 23%
	(ns)	(ns)	(*p* = 0.008)	(*p* = 0.002)	(ns)	(ns)	(ns)	(ns)	(ns)	(ns)

ExpSt—experimental standard diet with LNCaP and CtrlSt—control standard diet without LNCaP; ExpCu—experimental copper-supplemented diet with LNCaP and CtrlCu—control copper-supplemented diet without LNCaP; ExpSe—experimental selenium-supplemented diet with LNCa and CtrlSe—control selenium-supplemented diet without LNCaP; n—total number of rats. Comparative control to study on the same diets—* statistically significant (*p*-value above 0.05); ns—no statistically significant); ↓—decrease; ↑—increase.

## References

[1] Weatherholt A.M., Fuchs R.K., Warden S.J. (2012). Specialized Connective Tissue: Bone, the Structural Framework of the Upper Extremity. J. Hand Ther..

[2] Muselin F., Gârban Z., Cristina R.T., Doma A.O., Dumitrescu E., Vițălaru A.B., Bănățean-Dunea I. (2019). Homeostatic Changes of Some Trace Elements in Geriatric Rats in the Condition of Oxidative Stress Induced by Aluminum and the Beneficial Role of Resveratrol. J. Trace Elem. Med. Biol..

[3] Boskey A., Pleshko Camacho N. (2007). FT-IR Imaging of Native and Tissue-Engineered Bone and Cartilage. Biomaterials.

[4] Hart N.H., Nimphius S., Rantalainen T., Ireland A., Siafarikas A., Newton R.U. (2017). Mechanical Basis of Bone Strength: Influence of Bone Material, Bone Structure and Muscle Action. J. Musculoskelet. Neuronal Interact..

[5] Boskey A.L., Coleman R. (2010). Aging and Bone. J. Dent. Res..

[6] Robey P.G., Boskey A.L., Leikin S., Dempster D.W., Cauley J.A., Bouxsein M.L., Cosman F. (2021). Chapter 8—The regulatory role of matrix proteins in mineralization of bone. Marcus and Feldman’s Osteoporosis.

[7] Hernandez R.K., Wade S.W., Reich A., Pirolli M., Liede A., Lyman G.H. (2018). Incidence of Bone Metastases in Patients with Solid Tumors: Analysis of Oncology Electronic Medical Records in the United States. BMC Cancer.

[8] Lanocha N., Kalisinska E., Kosik-Bogacka D.I., Budis H., Sokolowski S., Bohatyrewicz A., Lanocha A. (2013). The Effect of Environmental Factors on Concentration of Trace Elements in Hip Joint Bones of Patients after Hip Replacement Surgery. Ann. Agric. Environ. Med..

[9] Brodziak-Dopierala B., Kwapulinski J., Kusz D., Gajda Z., Sobczyk K. (2009). Interactions between Concentrations of Chemical Elements in Human Femoral Heads. Arch. Environ. Contam. Toxicol..

[10] Kubaszewski Ł., Zioła-Frankowska A., Gasik Z., Frankowski M., Dąbrowski M., Molisak B., Kaczmarczyk J., Gasik R. (2017). Chemometric Evaluation of Concentrations of Trace Elements in Intervertebral Disc Tissue in Patient with Degenerative Disc Disease. Ann. Agric. Environ. Med..

[11] Bi X., Sterling J.A., Merkel A.R., Perrien D.S., Nyman J.S., Mahadevan-Jansen A. (2013). Prostate Cancer Metastases Alter Bone Mineral and Matrix Composition Independent of Effects on Bone Architecture in Mice—A Quantitative Study Using MicroCT and Raman Spectroscopy. Bone.

[12] Syniachenko O.V., Dumanckyi Y.V., Yehudina Y.D., Bevzenko T.B., Yarmola T.I. (2018). Bone Tissue Lesion in Oncological Disease (Literature Review and Own Research Data). Wiadomości Lek..

[13] Pepa G.D., Brandi M.L. (2016). Microelements for Bone Boost: The Last but Not the Least. Clin. Cases Miner. Bone Metab..

[14] Dermience M., Lognay G., Mathieu F., Goyens P. (2015). Effects of Thirty Elements on Bone Metabolism. J. Trace Elem. Med. Biol..

[15] Denoyer D., Clatworthy S.A.S., Masaldan S., Meggyesy P.M., Cater M.A. (2015). Heterogeneous Copper Concentrations in Cancerous Human Prostate Tissues. Prostate.

[16] Zeng H., Cao J.J., Combs G.F. (2013). Selenium in Bone Health: Roles in Antioxidant Protection and Cell Proliferation. Nutrients.

[17] Hoeg A., Gogakos A., Murphy E., Mueller S., Köhrle J., Reid D.M., Glüer C.C., Felsenberg D., Roux C., Eastell R. (2012). Bone Turnover and Bone Mineral Density Are Independently Related to Selenium Status in Healthy Euthyroid Postmenopausal Women. J. Clin. Endocrinol. Metab..

[18] Cai X., Wang C., Yu W., Fan W., Wang S., Shen N., Wu P., Li X., Wang F. (2016). Selenium Exposure and Cancer Risk: An Updated Meta-Analysis and Meta-Regression. Sci. Rep..

[19] Beukhof C.M., Medici M., van den Beld A.W., Hollenbach B., Hoeg A., Visser W.E., de Herder W.W., Visser T.J., Schomburg L., Peeters R.P. (2016). Selenium Status Is Positively Associated with Bone Mineral Density in Healthy Aging European Men. PLoS ONE.

[20] Kubiak K., Klimczak A., Dziki Ł., Modranka R., Malinowska K. (2010). Influence of copper (II) complex on the activity of selected oxidative enzymes. Pol. Merkur. Lek. Organ Pol. Tow. Lek..

[21] Li B., Yu S. (2007). In vitro study of the effects of copper ion on osteoclastic resorption in various dental mineralized tissues. Zhonghua Kou Qiang Yi Xue Za Zhi Zhonghua Kouqiang Yixue Zazhi Chin. J. Stomatol..

[22] Gaetke L.M., Chow-Johnson H.S., Chow C.K. (2014). Copper: Toxicological Relevance and Mechanisms. Arch. Toxicol..

[23] Burkhead J.L., Gray L.W., Lutsenko S. (2011). Systems Biology Approach to Wilson’s Disease. Biometals.

[24] de Romaña D.L., Olivares M., Uauy R., Araya M. (2011). Risks and Benefits of Copper in Light of New Insights of Copper Homeostasis. J. Trace Elem. Med. Biol..

[25] Golabek T., Darewicz B., Borawska M., Socha K., Markiewicz R., Kudelski J. (2012). Copper, Zinc, and Cu/Zn Ratio in Transitional Cell Carcinoma of the Bladder. Urol. Int..

[26] Malavolta M., Piacenza F., Basso A., Giacconi R., Costarelli L., Mocchegiani E. (2015). Serum Copper to Zinc Ratio: Relationship with Aging and Health Status. Mech. Ageing Dev..

[27] Mandair D., Rossi R.E., Pericleous M., Whyand T., Caplin M.E. (2014). Prostate Cancer and the Influence of Dietary Factors and Supplements: A Systematic Review. Nutr. Metab..

[28] Goodman V.L., Brewer G.J., Merajver S.D. (2004). Copper Deficiency as an Anti-Cancer Strategy. Endocr. Relat. Cancer.

[29] Liu H., Bian W., Liu S., Huang K. (2012). Selenium Protects Bone Marrow Stromal Cells against Hydrogen Peroxide-Induced Inhibition of Osteoblastic Differentiation by Suppressing Oxidative Stress and ERK Signaling Pathway. Biol. Trace Elem. Res..

[30] Rayman M.P. (2009). Selenoproteins and Human Health: Insights from Epidemiological Data. Biochim. Biophys. Acta.

[31] Mody N., Parhami F., Sarafian T.A., Demer L.L. (2001). Oxidative stress modulates osteoblastic differentiation of vascular and bone cells. Free Radic. Biol. Med..

[32] Hiraoka K., Komiya S., Hamada T., Zenmyo M., Inoue A. (2001). Osteosarcoma cell apoptosis induced by selenium. J. Orthop. Res..

[33] Yang T., Lee S.J., Park K.C., Park S.H., Chung J., Lee S. (2022). The Effects of Selenium on Bone Health: From Element to Therapeutics. Molecules.

[34] Rogers A., Eastell R. (2005). Circulating osteoprotegerin and receptor activator for nuclear factor (B ligand: Clinical utility in metabolic bone disease assessment. J. Clin. Endocrinol. Metab..

[35] Hofbauer L.C., Schoppet M. (2004). Clinical implications of the osteoprotegerin/RANKL/RANK system for bone and vascular diseases. JAMA.

[36] Guo D., Keightley A., Guthrie J., Veno P.A., Harris S.E., Bonewald L.F. (2010). Identification of osteocyte-selective proteins. Proteomics.

[37] Moon H.J., Ko W.K., Han S.W., Kim D.S., Hwang Y.S., Park H.K., Kwon I.K. (2012). Antioxidants, like coenzyme Q10, selenite, and curcumin, inhibited osteoclast differentiation by suppressing reactive oxygen species generation. Biochem. Biophys. Res. Commun..

[38] Ebert R., Ulmer M., Zeck S., Meissner-Weigl J., Schneider D., Stopper H., Schupp N., Kassem M., Jakob F. (2006). Selenium supplementation restores the antioxidative capacity and prevents cell damage in bone marrow stromal cells in vitro. Stem Cells.

[39] Cao J.J., Gregoire B.R., Zeng H. (2012). Selenium Deficiency Decreases Antioxidative Capacity and Is Detrimental to Bone Microarchitecture in Mice. J. Nutr..

[40] Zhang J., Munger R.G., West N.A., Cutler D.R., Wengreen H.J., Corcoran C.D. (2006). Antioxidant Intake and Risk of Osteoporotic Hip Fracture in Utah: An Effect Modified by Smoking Status. Am. J. Epidemiol..

[41] Wang X., Ning Y., Yang L., Yu F., Guo X. (2017). Zinc: The Other Suspected Environmental Factor in Kashin-Beck Disease in Addition to Selenium. Biol. Trace Elem. Res..

[42] Mlakar S.J., Osredkar J., Prezelj J., Marc J. (2010). The Antioxidant Enzyme GPX1 Gene Polymorphisms Are Associated with Low BMD and Increased Bone Turnover Markers. Dis. Markers.

[43] Sun J., Sun Q., Lu S. (2011). From Selenoprotein to Endochondral Ossification: A Novel Mechanism with MicroRNAs Potential in Bone Related Diseases?. Med. Hypotheses.

[44] Bos S.D., Kloppenburg M., Suchiman E., van Beelen E., Slagboom P.E., Meulenbelt I. (2009). The Role of Plasma Cytokine Levels, CRP and Selenoprotein S Gene Variation in OA. Osteoarthr. Cartil..

[45] Wang N., Xie D., Wu J., Wu Z., He H., Yang Z., Yang T., Wang Y. (2020). Selenium and bone health: A protocol for a systematic review and metaanalysis. BMJ.

[46] Rivas A., Romero A., Mariscal-Arcas M., Monteagudo C., López G., Lorenzo M.L., Ocaña-Peinado F.M., Olea-Serrano F. (2012). Association between dietary antioxidant quality score (DAQs) and bone mineral density in Spanish women. Nutr. Hosp..

[47] Sun L.L., Li B.L., Xie H.L., Fan F., Yu W.Z., Wu B.H., Xue W.Q., Chen Y.M. (2014). Associations between the dietary intake of antioxidant nutrients and the risk of hip fracture in elderly Chinese: A case-control study. Br. J. Nutr..

[48] Melhus H., Michaëlsson K., Holmberg L., Wolk A., Ljunghall S. (1999). Smoking, antioxidant vitamins, and the risk of hip fracture. J. Bone Miner. Res..

[49] Wolf R.L., Cauley J.A., Pettinger M., Jackson R., Lacroix A., Leboff M.S., Lewis C.E., Nevitt M.C., Simon J.A., Stone K.L. (2005). Lack of a relation between vitamin and mineral antioxidants and bone mineral density: Results from the women’s health Initiative. Am. J. Clin. Nutr..

[50] Arikan D.C., Coskun A., Ozer A., Kilinc M., Atalay F., Arikan T. (2011). Plasma selenium, zinc, copper and lipid levels in postmenopausal Turkish women and their relation with osteoporosis. Biol. Trace Elem. Res..

[51] Ilich J.Z., Cvijetic S., Baric I.C., Cecic I., Saric M., Crncevic-Orlic Z., Blanusa M., Korsic M. (2009). Nutrition and lifestyle in relation to bone health and body weight in Croatian postmenopausal women. Int. J. Food Sci. Nutr..

[52] Wang L., Yu H., Yang G., Zhang Y., Wang W., Su T., Ma W., Yang F., Chen L., He L. (2015). Correlation between bone mineral density and serum trace element contents of elderly males in Beijing urban area. Int. J. Clin. Exp. Med..

[53] Liu S.-Z., Yan H., Xu P., Li J.-P., Zhuang G.-H., Zhu B.-F., Lu S.-M. (2009). Correlation analysis between bone mineral density and serum element contents of postmenopausal women in Xi’an urban area. Biol. Trace Elem. Res..

[54] Walsh J.S., Jacques R.M., Schomburg L., Hill T.R., Mathers J.C., Williams G.R., Eastell R. (2021). Effect of selenium supplementation on musculoskeletal health in older women: A randomised, double-blind, placebo-controlled trial. Lancet Healthy Longev..

[55] Perri G., Hill T., Mathers J.C., Walsh J., Gossiel F., Winther K., Frölich J., Folkestad L., Cold S., Eastell R. (2021). Effect of selenium supplementation on biomarkers of bone turnover. Proc. Nutr. Soc..

[56] Lippman S.M., Klein E.A., Goodman P.J., Lucia M.S., Thompson I.M., Ford L.G., Parnes H.L., Minasian L.M., Gaziano J.M., Hartline J.A. (2009). Effect of Selenium and Vitamin E on Risk of Prostate Cancer and Other Cancers: The Selenium and Vitamin E Cancer Prevention Trial (SELECT). JAMA J. Am. Med. Assoc..

[57] Stranges S., Marshall J.R., Natarajan R., Donahue R.P., Trevisan M., Combs G.F., Cappuccio F.P., Ceriello A., Reid M.E. (2007). Effects of Long-Term Selenium Supplementation on the Incidence of Type 2 Diabetes: A Randomized Trial. Ann. Intern. Med..

[58] Fulda S. (2011). Targeting apoptosis signaling pathways for anticancer therapy. Front. Oncol..

[59] Sinha K., Das J., Pal P.B., Sil P.C. (2013). Oxidative stress: The mitochondria dependent and mitochondria independent pathways of apoptosis. Arch. Toxicol..

[60] Aki T., Funakoshi T., Uemura K. (2015). Regulated necrosis and its implications in toxicology. Toxicology.

[61] Ju-Kun S., Yuan D.-B., Rao H.-F., Chen T.-F., Luan B.-S., Xu X.-M., Jiang F.-N., Zhong W.-D., Zhu J.-G. (2016). Association between Cd Exposure and Risk of Prostate Cancer. Medicine.

[62] Banas A., Kwiatek W.M., Banas K., Gajda M., Pawlicki B., Cichocki T. (2010). Correlation of Concentrations of Selected Trace Elements with Gleason Grade of Prostate Tissues. J. Biol. Inorg. Chem..

[63] Lener M., Jaworska K., Muszyńska M., Sukiennicki G., Durda K., Gupta S., Złowocka-Perłowska E., Kładny J., Wiechowska-Kozłowska A., Grodzki T. (2012). Selenium as Marker for Cancer Risk and Prevention. Pol. Przegl. Chir..

[64] Banerjee P.P., Banerjee S., Brown T.R., Zirkin B.R. (2018). Androgen Action in Prostate Function and Disease. Am. J. Clin. Exp. Urol..

[65] Menter D.G., Sabichi A.L., Lippman S.M. (2000). Selenium Effects on Prostate Cell Growth. Cancer Epidemiol. Biomark. Prev..

[66] Brozmanová J., Mániková D., Vlčková V., Chovanec M. (2010). Selenium: A Double-Edged Sword for Defense and Offence in Cancer. Arch. Toxicol..

[67] Uysal H., Agar G. (2005). Selenium Protective Activity against Aflatoxin B1 Adverse Affects on Drosophila Melanogaster. Braz. Arch. Biol. Technol..

[68] Rejali L., Jaafar M.H., Ismail N.H. (2007). Serum Selenium Level and Other Risk Factors for Breast Cancer among Patients in a Malaysian Hospital. Environ. Health Prev. Med..

[69] Sandsveden M., Manjer J. (2017). Selenium and Breast Cancer Risk: A Prospective Nested Case–Control Study on Serum Selenium Levels, Smoking Habits and Overweight. Int. J. Cancer.

[70] Liu D., Kuai Y., Zhu R., Zhou C., Tao Y., Han W., Chen Q. (2020). Prognosis of Prostate Cancer and Bone Metastasis Pattern of Patients: A SEER-Based Study and a Local Hospital Based Study from China. Sci. Rep..

[71] Zhang X.H.-F., Jin X., Malladi S., Zou Y., Wen Y.H., Brogi E., Smid M., Foekens J., Massagué J. (2013). Selection of Bone Metastasis Seeds by Mesenchymal Signals in the Primary Tumor Stroma. Cell.

[72] Weilbaecher K.N., Guise T.A., McCauley L.K. (2011). Cancer to Bone: A Fatal Attraction. Nat. Rev. Cancer.

[73] Dushyanthen S., Cossigny D.A.F., Quan G.M.Y. (2013). The Osteoblastic and Osteoclastic Interactions in Spinal Metastases Secondary to Prostate Cancer. Cancer Growth Metastasis.

[74] Zhu X.-C., Zhang J.-L., Ge C.-T., Yu Y.-Y., Wang P., Yuan T.-F., Fu C.-Y. (2015). Advances in Cancer Pain from Bone Metastasis. Drug Des. Dev. Ther..

[75] Ando T., Watanabe K., Mizusawa T., Katagiri A. (2018). Hypercalcemia Due to Parathyroid Hormone-Related Peptide Secreted by Neuroendocrine Dedifferentiated Prostate Cancer. Urol. Case Rep..

[76] Cannarella R., Condorelli R.A., Barbagallo F., La Vignera S., Calogero A.E. (2021). Endocrinology of the Aging Prostate: Current Concepts. Front. Endocrinol..

[77] Vuichoud C., Loughlin K.R. (2015). Benign Prostatic Hyperplasia: Epidemiology, Economics and Evaluation. Can. J. Urol..

[78] Daniyal M., Siddiqui Z.A., Akram M., Asif H.M., Sultana S., Khan A. (2014). Epidemiology, Etiology, Diagnosis and Treatment of Prostate Cancer. Asian Pac. J. Cancer Prev. APJCP.

[79] Cancer Today. http://gco.iarc.fr/today/home.

[80] Siegel R.L., Miller K.D., Jemal A. (2016). Cancer Statistics. CA Cancer J. Clin..

[81] Keller E.T., Zhang J., Cooper C.R., Smith P.C., McCauley L.K., Pienta K.J., Taichman R.S. (2001). Prostate Carcinoma Skeletal Metastases: Cross-Talk between Tumor and Bone. Cancer Metastasis Rev..

[82] Wong S.K., Mohamad N.-V., Giaze T.R., Chin K.-Y., Mohamed N., Ima-Nirwana S. (2019). Prostate Cancer and Bone Metastases: The Underlying Mechanisms. Int. J. Mol. Sci..

[83] Gaffney-Stomberg E. (2019). The Impact of Trace Minerals on Bone Metabolism. Biol. Trace Elem. Res..

[84] Noor Z., Sumitro S.B., Hidayat M., Rahim A.H., Sabarudin A., Umemura T. (2012). Atomic Mineral Characteristics of Indonesian Osteoporosis by High-Resolution Inductively Coupled Plasma Mass Spectrometry. Sci. World J..

[85] Jonas J., Burns J., Abel E.W., Cresswell M.J., Strain J.J., Paterson C.R. (1993). Impaired Mechanical Strength of Bone in Experimental Copper Deficiency. Ann. Nutr. Metab..

[86] Rondanelli M., Faliva M.A., Infantino V., Gasparri C., Iannello G., Perna S., Riva A., Petrangolini G., Tartara A., Peroni G. (2021). Copper as Dietary Supplement for Bone Metabolism: A Review. Nutrients.

[87] Milkovic L., Hoppe A., Detsch R., Boccaccini A.R., Zarkovic N. (2014). Effects of Cu-Doped 45S5 Bioactive Glass on the Lipid Peroxidation-Associated Growth of Human Osteoblast-like Cells in Vitro. J. Biomed. Mater. Res. A.

[88] National Research Council (US) (1995). Subcommittee on Laboratory Animal Nutrition. Nutrient Requirements of Laboratory Animals: Fourth Revised Edition.

[89] Ott G., Havemeyer A., Clement B. (2015). The Mammalian Molybdenum Enzymes of MARC. J. Biol. Inorg. Chem. JBIC.

[90] Lewis R.C., Johns L.E., Meeker J.D. (2016). Exploratory Analysis of the Potential Relationship between Urinary Molybdenum and Bone Mineral Density among Adult Men and Women from NHANES 2007–2010. Chemosphere.

[91] Vyskocil A., Viau C. (1999). Assessment of Molybdenum Toxicity in Humans. J. Appl. Toxicol. JAT.

[92] Bhattacharya P.T., Misra S.R., Hussain M. (2016). Nutritional Aspects of Essential Trace Elements in Oral Health and Disease: An Extensive Review. Scientifica.

[93] Parry N.M., Phillippo M., Reid M.D., McGaw B.A., Flint D.J., Loveridge N. (1993). Molybdenum-Induced Changes in the Epiphyseal Growth Plate. Calcif. Tissue Int..

[94] Picco S., Ponzzinibio M.V., Mattioli G., Rosa D., Minatel L., Fazzio L., Seoane A. (2012). Physiological and Genotoxic Effects of Molybdenum-Induced Copper Deficiency in Cattle. Agrociencia.

[95] Soetan K.O., Olaiya C.O., Oyewole O.E. (2010). The Importance of Mineral Elements for Humans, Domestic Animals and Plants—A Review. Afr. J. Food Sci..

[96] Aschner J.L., Aschner M. (2005). Nutritional Aspects of Manganese Homeostasis. Mol. Asp. Med..

[97] Sansone V., Pagani D., Melato M. (2013). The Effects on Bone Cells of Metal Ions Released from Orthopaedic Implants. A Review. Clin. Cases Miner. Bone Metab..

[98] Devitt B.M., Queally J.M., Vioreanu M., Butler J.S., Murray D., Doran P.P., O’Byrne J.M. (2010). Cobalt Ions Induce Chemokine Secretion in a Variety of Systemic Cell Lines. Acta Orthop..

[99] Gibon E., Amanatullah D.F., Loi F., Pajarinen J., Nabeshima A., Yao Z., Hamadouche M., Goodman S.B. (2017). The Biological Response to Orthopaedic Implants for Joint Replacement: Part I: Metals. J. Biomed. Mater. Res. B Appl. Biomater..

[100] Queally J.M., Devitt B.M., Butler J.S., Malizia A.P., Murray D., Doran P.P., O’Byrne J.M. (2009). Cobalt Ions Induce Chemokine Secretion in Primary Human Osteoblasts. J. Orthop. Res..

[101] Tkaczyk C., Petit A., Antoniou J., Zukor D.J., Tabrizian M., Huk O.L. (2010). Significance of Elevated Blood Metal Ion Levels in Patients with Metal-on-Metal Prostheses: An Evaluation of Oxidative Stress Markers. Open Orthop. J..

[102] Tkaczyk C., Huk O.L., Mwale F., Antoniou J., Zukor D.J., Petit A., Tabrizian M. (2010). Effect of Chromium and Cobalt Ions on the Expression of Antioxidant Enzymes in Human U937 Macrophage-like Cells. J. Biomed. Mater. Res. A.

[103] Salminen A., Kauppinen A., Kaarniranta K. (2015). 2-Oxoglutarate-Dependent Dioxygenases Are Sensors of Energy Metabolism, Oxygen Availability, and Iron Homeostasis: Potential Role in the Regulation of Aging Process. Cell. Mol. Life Sci..

[104] Díaz-Castro J., López-Frías M.R., Campos M.S., López-Frías M., Alférez M.J.M., Nestares T., Ojeda M.L., López-Aliaga I. (2012). Severe Nutritional Iron-Deficiency Anaemia Has a Negative Effect on Some Bone Turnover Biomarkers in Rats. Eur. J. Nutr..

[105] Jeney V. (2017). Clinical Impact and Cellular Mechanisms of Iron Overload-Associated Bone Loss. Front. Pharmacol..

[106] Strause L., Saltman P., Smith K.T., Bracker M., Andon M.B. (1994). Spinal Bone Loss in Postmenopausal Women Supplemented with Calcium and Trace Minerals. J. Nutr..

[107] Eaton-Evans J., Mcllrath E.M., Jackson W.E., McCartney H., Strain J.J. (1996). Copper Supplementation and the Maintenance of Bone Mineral Density in Middle-Aged Women. J. Trace Elem. Exp. Med..

[108] Baker A., Harvey L., Majask-Newman G., Fairweather-Tait S., Flynn A., Cashman K. (1999). Effect of Dietary Copper Intakes on Biochemical Markers of Bone Metabolism in Healthy Adult Males. Eur. J. Clin. Nutr..

[109] Nielsen F.H., Lukaski H.C., Johnson L.K., Roughead Z.K.F. (2011). Reported Zinc, but Not Copper, Intakes Influence Whole-Body Bone Density, Mineral Content and T Score Responses to Zinc and Copper Supplementation in Healthy Postmenopausal Women. Br. J. Nutr..

[110] Jomova K., Valko M. (2011). Advances in Metal-Induced Oxidative Stress and Human Disease. Toxicology.

[111] Yossepowitch O., Pinchuk I., Gur U., Neumann A., Lichtenberg D., Baniel J. (2007). Advanced but Not Localized Prostate Cancer Is Associated with Increased Oxidative Stress. J. Urol..

[112] Paschos A., Pandya R., Duivenvoorden W.C.M., Pinthus J.H. (2013). Oxidative Stress in Prostate Cancer: Changing Research Concepts towards a Novel Paradigm for Prevention and Therapeutics. Prostate Cancer Prostatic Dis..

[113] Ayala G.E., Dai H., Ittmann M., Li R., Powell M., Frolov A., Wheeler T.M., Thompson T.C., Rowley D. (2004). Growth and Survival Mechanisms Associated with Perineural Invasion in Prostate Cancer. Cancer Res..

[114] Vogt T.M., Ziegler R.G., Graubard B.I., Swanson C.A., Greenberg R.S., Schoenberg J.B., Swanson G.M., Hayes R.B., Mayne S.T. (2003). Serum Selenium and Risk of Prostate Cancer in U.S. Blacks and Whites. Int. J. Cancer.

[115] Cui Z., Liu D., Liu C., Liu G. (2017). Serum Selenium Levels and Prostate Cancer Risk: A MOOSE-Compliant Meta-Analysis. Medicine.

[116] Sayehmiri K., Azami M., Mohammadi Y., Soleymani A., Tardeh Z. (2018). The Association between Selenium and Prostate Cancer: A Systematic Review and Meta-Analysis. Asian Pac. J. Cancer Prev. APJCP.

[117] Algotar A.M., Stratton M.S., Ahmann F.R., Ranger-Moore J., Nagle R.B., Thompson P.A., Slate E., Hsu C.H., Dalkin B.L., Sindhwani P. (2013). Phase 3 Clinical Trial Investigating the Effect of Selenium Supplementation in Men at High-Risk for Prostate Cancer. Prostate.

[118] Klein E.A., Thompson I.M., Tangen C.M., Crowley J.J., Lucia M.S., Goodman P.J., Minasian L.M., Ford L.G., Parnes H.L., Gaziano J.M. (2011). Vitamin E and the Risk of Prostate Cancer: The Selenium and Vitamin E Cancer Prevention Trial (SELECT). JAMA.

[119] Nicastro H.L., Dunn B.K. (2013). Selenium and Prostate Cancer Prevention: Insights from the Selenium and Vitamin E Cancer Prevention Trial (SELECT). Nutrients.

[120] Bhaskaram P. (2002). Micronutrient Malnutrition, Infection, and Immunity: An Overview. Nutr. Rev..

[121] Zeng H. (2002). Selenite and Selenomethionine Promote HL-60 Cell Cycle Progression. J. Nutr..

[122] Saito Y., Yoshida Y., Akazawa T., Takahashi K., Niki E. (2003). Cell Death Caused by Selenium Deficiency and Protective Effect of Antioxidants. J. Biol. Chem..

[123] Karp H.J., Ketola M.E., Lamberg-Allardt C.J.E. (2009). Acute Effects of Calcium Carbonate, Calcium Citrate and Potassium Citrate on Markers of Calcium and Bone Metabolism in Young Women. Br. J. Nutr..

[124] Sakhaee K., Maalouf N.M., Abrams S.A., Pak C.Y.C. (2005). Effects of Potassium Alkali and Calcium Supplementation on Bone Turnover in Postmenopausal Women. J. Clin. Endocrinol. Metab..

[125] Akbal A., Yılmaz H., Tutkun E. (2014). Arsenic Exposure Associated with Decreased Bone Mineralization in Male. Aging Male.

[126] Richette P., Ottaviani S., Vicaut E., Bardin T. (2010). Musculoskeletal Complications of Hereditary Hemochromatosis: A Case-Control Study. J. Rheumatol..

[127] Brissot P., Cavey T., Ropert M., Guggenbuhl P., Loréal O. (2017). Genetic Hemochromatosis: Pathophysiology, Diagnostic and Therapeutic Management. Presse Med..

[128] Kim B.-J., Ahn S.H., Bae S.J., Kim E.H., Lee S.-H., Kim H.-K., Choe J.W., Koh J.-M., Kim G.S. (2012). Iron Overload Accelerates Bone Loss in Healthy Postmenopausal Women and Middle-Aged Men: A 3-Year Retrospective Longitudinal Study. J. Bone Miner. Res..

